# Information Literacy as a Predictor of Work Performance: The Mediating Role of Lifelong Learning and Creativity

**DOI:** 10.3390/bs13010024

**Published:** 2022-12-26

**Authors:** Muhammad Asif Naveed, Javed Iqbal, Muhammad Zaheer Asghar, Rozeen Shaukat, Pirita Seitamaa-hakkarainen

**Affiliations:** 1 Department of Information Management, University of Sargodha, Sargodha 40100, Pakistan; 2 School of Education, Huazhong University of Science and Technology, Wuhan 430074, China; 3 Faculty of Management Sciences, Ilma University Karachi, Karachi 75190, Pakistan; 4 Department of Teacher Education, University of Helsinki, 00014 Helsinki, Finland; 5 Office of Research Innovation and Commercialization, University of Management and Technology, Lahore 54770, Pakistan

**Keywords:** workplace information literacy, lifelong learning, creativity, work performance, journalists, Pakistan

## Abstract

This study examined the effect of information literacy (IL) on work performance with mediating role of lifelong learning and creativity among journalists in Pakistan. A cross-sectional survey using an online questionnaire was conducted in the press clubs of four provinces (e.g., Punjab, Sindh, Khyber Pakhtunkhwa, and Baluchistan) and the federal capital Islamabad for data collection. The received 1084 responses were analyzed using the partial least squares structural equation modelling. The results indicated that IL of journalists had a direct and indirect but positive influence on their work performance. The lifelong learning and creativity skills also mediated the relationship between IL and work performance. This study provided empirical evidence for how IL directly influence work performance and indirectly with the mediated role of lifelong learning and creativity. These pragmatic insights may inform academicians and enterprises about the IL importance at workplace for enhancement of organizational performance and achieving a competitive advantage. Such results may also initiate an instruction program for existing as well as for prospective journalists to impart IL education. This study could be a worthy contribution to the existing IL research in the workplace context in general and of journalists’ workplace in particular as no such study has appeared so far.

## 1. Introduction

Information literacy (IL) has been recognized as an essential set of capabilities enabling enterprises to leverage information assets efficiently and effectively for having a business value [1] innovation [2] and sustainable competitive advantage [3,4,5]. Previous research in the context of workplace proposed that IL informs organizations in critical market analysis, making balanced judgments, and implementing effective strategies contributing towards sustained growth and competitiveness [2,6,7]. The IL value creation is dependent on how well workforce perform in the workplace, particularly in environmental scanning, managing information, and research and development and the quality of information, follow [8]. IL builds the capacity of the workforce in undertaking information-related tasks, making the best information use along with its judicious interpretations and adopting an appropriate information behavior, and making an informed decisions to achieve organization goals [9,10,11]. Therefore, the corporations needed to become information literate to monitor new, unique, and prevailing trends in the market and develop such products which had potential to meet market needs. Despite the value of IL in the business workplace, relatively limited empirical evidence has appeared so far supporting the influence of IL on organizational and work performance [12]. Work performance has been defined as the workers’ consistent behavior contributing towards organizational mission and generating cumulative business value to the corporations [13,14]. The solid performance of the workers has the potential to improve organizational performance and effectives. Therefore, the promotion of work performance has become one of the fundamental issues that modern enterprises face. The learning of new knowledge, skills, and expertise by the employees enhances their job performance in the workplace [15].

The pursuit of knowledge and skills acquisition which is ongoing, voluntary, and self-initiated is labeled as lifelong learning [16]. Lifelong learning is crucial for creation and maintenance of positive attitudes among employees toward knowledge acquisition and application either for personal or professional development [17]. Creativity refers to an aptitude to produce new, distinctive, valuable, and original ideas to achieve a sustainable competitive advantage [18]. Thus, creative workers are essential to achieving organizational goals [19]. Ref. [20] highlighted the importance of integrating creativity into education and learning. Interestingly, continuous and creative learning is beneficial for not only employees but also for employers [21]. Both lifelong learning and creativity undertakes certain information-related tasks and are dependent on appropriate information behaviors. Some inquiries have reported that IL capabilities can improve lifelong learning [22], creativity [12,23] and innovative work behaviors [24], while theorizing points to a potentially positive effects of IL on lifelong learning, creativity, and work performance, clear empirical evidence is still lacking. Since the existing empirical evidence is still limited, the interrelationships of IL, lifelong learning, creativity, and work performance merits further investigation.

In view of the above discussion, this research intended to empirically investigate the relationship between IL, lifelong learning, creativity, and work performance among journalists in Pakistan. The reason for selection of journalists as a unit of analysis was that the job of a journalist is diverse, time critical, and research intensive as they need, consume, create, and distribute information simultaneously [25]. Therefore, the journalist requires the adoption of an appropriate information behavior for writing investigative stories, creating news items, and framing opinion pieces, and needs to have a special set of IL skills to avoid information disorders [26,27,28]. In addition, there were only a few studies addressing IL in the journalists’ workplace globally [27,28]. Understanding the interrelationships of IL, lifelong learning, creativity, and work performance in the journalists’ workplace would help organizations in recognizing how IL influence in the workplace. The present research has specifically addressed the following research questions:

Research Questions (RQs):

1. How does information literacy influence work performance?
2. How does information literacy influence lifelong learning and creativity?
3. How do lifelong learning and creativity influence work performance?
4. How do lifelong learning and creativity mediate the relationship between information literacy and work performance?

### 1.1. Literature Review and Research Hypotheses

#### 1.1.1. Workplace Information Literacy (WIL)

With the voluminous growth of information and the realization that the organization must have to deal with it, the information literate workforce is essential to achieve a sustainable competitive advantage. Therefore, IL research in workplace context has not only witnessed growth in the last decade but also gaining a phenomenal momentum [1,2,11]. Of the seminal works, Ref. [29] addressed IL in the university workplace using researchers, IT professionals, and counsellors and identified IL as a fundamental characteristic of learning organizations, while the study by [30] identified IL as a stimulating and key aspect of knowledge creation at law firms enabling employees in recognition and seeking of needed information and making sense of it. Ref. [31] reported that the senior managers perceived IL as a catalyst for information flow, innovation, decision making, and influencing others. Likewise, firefighters and ambulance workers consider IL as a key factor for learning, navigating with an everchanging information landscape and developing social networks [32]. While describing an example of a leading environmental consulting company of the world, Ref. [33] recognized IL as a vital component of knowledge management program to enhance the organizational value.

Ref [34] conceptualized IL as driver for informed learning in the workplace enabling collaborative use of information in a socio-technical system. Moreover, the web professionals experienced IL as staying informed, developing successful websites, solving problems, and engaging with learning communities [2]. According to [35], many employers may not have an appropriate understanding of their employees’ IL levels. Resultantly, both employers and employees “may not know that they don’t know” (p. 93). In another study, Ref. [1] considers IL as learning, experienced as task-focused information need having its fulfilment through effective information engagement. Further, Ref. [36] described certain key IL benefits: improved operational efficiency, informed decision making, reduced time, increased profitability, improved customer service, enhanced motivation, and maximized information governance. The content analysis of job advertisements for entry-level advertising professionals by [37] also identified that the expertise in utilization and evaluation of information resources, team collaboration, and technological competence were mentioned in at least 41% of the job advertisement. Thus, IL is an essential expertise in the workplace creating positive outcomes for organizations and increase its efficiency, effectiveness, and business opportunities [38,39,40,41].

This existing research, as a whole, has significantly enhanced understanding of WIL. There were many studies exploring IL emergence and experiences in the workplace and the research providing empirical evidences for the effects of WIL has remained scanty. In this regard, the study of [36] proposed a tool for understanding the IL value for enterprises towards work efficiency, profitability, customer service, staff motivation and information governance, while [42] proposed a WIL model having four stages, namely, share knowledge, task analysis, task execution, and evaluation. An inquiry by [2] not only developed a WIL scale but also investigated the impact of WIL on organization innovation using 184 CEOs of different companies. The SEM analysis indicated not only a direct and positive IL effect on the development of exploratory and exploitative innovations but also an indirect effect with the mediating role of opportunity recognition. A recent study by [28] investigated the IL impact on personal information management (PIM) practices among e-media reporters and reported IL as a positive predictor of PIM practices. Ref. [43] reviewed literature published form South Asia and reported the dearth of WIL literature produced by the region.

#### 1.1.2. IL and Work Performance

Although the positive relationship between IL and work performance are well established, few studies provide empirical evidence. Of those studies, Ref. [24] reported the positive role of IL in developing innovative work behaviors and work performance. Furthermore, IL not only has persistent relation with the individual job fit but also impacts positively work performance [44,45,46]. There was a positive association between IL and work performance of teachers, researchers, and knowledge workers [41]. In light of the ongoing discussion, it is inferred that IL skills increase work performance in varied workplace contexts. No study has appeared so far to report such association in the journalists’ workplace as the journalist primarily does information business. Therefore, the following research hypothesis was proposed:

**Hypothesis** **1.** *IL positively influences work performance.*

#### 1.1.3. IL and Lifelong Learning

The relationship of IL with lifelong learning has been under discussion since last two decades [39,47] as it overcomes the barriers to the lifelong learning process [29,48]. Some empirical studies reported that IL competencies enhance lifelong learning approaches [16,34,49]. Keeping this in mind, it was quite legitimate to empirically investigate the relationship of IL with lifelong learning in the journalists’ workplace. Thus, following hypothesis was formulated:

**Hypothesis** **2.** *IL positively influences lifelong learning.*

#### 1.1.4. IL and Creativity

The production of original and valuable ideas requires people to have the capability to find, access, evaluate, and manage information efficiently and effectively. The ability for recognition of information needs and seeking enables people to make judicious judgment on the applicability of information to creative task in hand [50]. Considering this viewpoint, it is quite logical to infer that IL may positively influence creativity as information literate individuals confidently navigate with contemporary information environment. A few studies from the workplace context also reported a positive effect of IL on creativity [12,23,51,52]. Conclusively, the related studies were limited, the relationship of IL with creativity merits further investigation. Hence, the following research hypothesis was developed:

**Hypothesis** **3.** *IL positively influences creativity.*

#### 1.1.5. Lifelong Learning and Work Performance

The lifelong learning skills of employees are also associated with work performance [53,54]. The study by [55] reported that the concept of learning organizations positively predicted work performance. This relationship merits further investigation as it needed to be strengthened with more evidence from the workplace. Hence, the following hypothesis was formulated:

**Hypothesis** **4.** *Lifelong learning positively influences work performance.*

#### 1.1.6. Creativity and Work Performance

Creativity produces new, valuable, and distinctive ideas for innovation and work performance [56]. The positive relationship between creativity and work performance was also reported by some previous studies [12,27,57,58,59]. The results of existing studies needed to be corroborated for theorization. Thus, the relationship between creativity and work performance justifies further investigation and the following hypothesis posited:

**Hypothesis** **5.** *Creativity positively influences work performance.*

#### 1.1.7. IL and Work Performance: Mediating Role of Lifelong Learning and Creativity

There was positive relationship of IL with work performance [24,44] and lifelong learning [34,49]. Lifelong learning also appeared to predict work performance positively [53,54,60]. The concepts of IL and lifelong learning had a strategic, mutual, and reinforcing relationship with one another. These two concepts are largely self-motivated, self-directed, self-empowered, and self-actuating, which can enhance the set of personal choices and improve the quality of learning and effective socio-economic participation [61]. IL lays the foundation for lifelong learning. Succinctly, both concepts are essential for the success of individuals, organizations, and societies if harnessed together particularly in the working environment [62]. Considering the three-way association between IL, lifelong learning, and work performance, the following hypothesis was formulated:

**Hypothesis** **6.** *Lifelong learning mediates the relationship between IL and work performance.*

A perusal of the published research also indicated that IL and creativity skills of employees directly influence their work performance [24,58,59]. The study by [12] reported that IL did not directly influence work performance but with the mediating role of creativity. In line with this three-way association, the mediating role of creativity between IL and work performance is predicted. Some other studies also reported the mediation role of creativity between the relationships of employee engagement and intrinsic motivation with job performance [63,64]. Thus, the following hypothesis was postulated:

**Hypothesis** **7.** *Creativity mediates the relationship between IL and work performance.*

## 2. Methodology

### 2.1. Research Model

**Figure 1:**
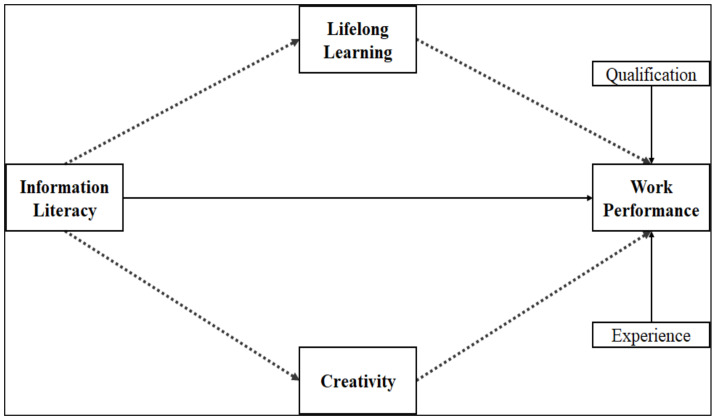
Proposed research model.

This research intended to examine the inter-relationship of IL, lifelong learning, creativity, and work performance among journalists as presented in the proposed research model which delineates the hypothesized relationships between these constructs (Figure 1).

### 2.2. Design and Method

This study deployed quantitative research design along with survey methods for examining the interrelationships between IL, lifelong learning, creativity, and work performance among journalists in Pakistan. The data were collected using a structured questionnaire as it was useful to collect responses from a geographically dispersed population. The questionnaire consisted of 38 items related to information literacy (13 items), lifelong learning (8 items), creativity (10 items), and work performance (7 items) along with demographic variables such as age, gender, qualification, experience, and region. Each item was measure on a five-point Likert Scale ranging from strongly agree to strongly disagree (e.g., 5 = strongly agree, 4 = agree, 3 = neither agree nor disagree, 2 = disagree, and 1 = strongly disagree).

### 2.3. Measures

#### 2.3.1. Information Literacy

There were 13 items related to IL generated based on standards for information literacy for journalists developed by the Association of College and Research Libraries (ACRL) in 2012. Examples such items include “I am able to identify the audience of my story” and “I am able to judge the content of the selected information sources”. The Cronbach alpha (CA) and composite reliability (CR) for the IL measure were 0.91 and 0.93, respectively. The value of CA varies between 0 and 1. The CA value near to one indicates higher internal consistency and near to zero indicates lower reliability [65]. The CR value is calculated as an alternative to CA due to criticism on CA for its lower bound value underestimating the true reliability as CR value is slightly higher than CA whereby the difference is relatively inconsequential [66].

#### 2.3.2. Lifelong Learning

The lifelong learning of these journalists was measured using 8-items adopted from the work of [67]. Examples of such items include “I would like to be active in my work for many years”, and “I feel very personally motivated in my work”. The figures in Table 2 indicated the reasonable and acceptable values of CA and CR for the measure of lifelong learning as 0.86 and 0.89, respectively, and CR is slightly higher than CA (Table 2).

#### 2.3.3. Creativity

Ten items related to creativity were adopted from creativity scale developed by [68]. Examples of such items include “I have the ability to solve problems which find difficult”, and “I usually find new uses for existing methods or existing equipment”. Table 2 shows high values for CA = 0.89 and CR = 0.91.

#### 2.3.4. Work Performance

Eight items were adopted from an individual work performance scale developed by [69] to the assess work performance of these journalists. Examples of such items include “I take on challenging tasks when they are available”, and “I come up with creative solutions for new problems”. The CA and CR values for the measure of work performance were 0.84 and 0.88, respectively (Table 2).

### 2.4. Population and Sampling

All the journalists with membership of press clubs of four provinces (e.g., Punjab, Sindh, Khyber Pakhtunkhwa, and Baluchistan) and federal capital (e.g., Islamabad) were considered as the study population. According to the representatives of each press, there were a total of 9512 registered members in all the press clubs, namely, Karachi Press Club (2005 members), Lahore Press Club (3050), Peshawar Press Club (550 members), Quetta Press Club (157 members), and National Press Club Islamabad (3750 members). The minimum sample size was 370 which was calculated based on 95% confidence interval and 5% margin of error using an online calculator of SurveyMonkey. The recruitment of the survey participants from each press club was made through stratified convenient sampling process due to time limitation and accessibility issues.

### 2.5. Data Collection

The data were collected from the journalists in Pakistan using an online questionnaire which was developed in Google Forms. The researchers shared the link to an online questionnaire along with a covering letter in listserves and WhatsApp groups of each press club by request to the respective administration. The journalists were asked to fill the online questionnaire voluntarily. These journalists were also assured of the confidentiality and anonymity of their responses. Initially, there was a quite low response rate. Multiple follow-up reminders were also sent to these journalists through listserves and WhatsApp groups in order to increase response rate. In addition, the researchers also approached different media houses and representatives of each press club through personal contacts and representatives of each press club were also asked for help in data collection from the registered members. A total of 1084 responses were received in approximately 3–4 months of data collection. The received responses were carefully screened before data analysis.

### 2.6. Data Analysis

Partial least squares structural equation modeling (PLS-SEM) was utilized to measure the direct and indirect relations between IL, lifelong learning, creativity, and work performance in the proposed research model through SmartPLS 3.3.3 version. Firstly, the reliability and validity were analyzed through measurement modeling such as factor loading, Cronbach Alpha, roh_A, and convergent validity and discriminant validity of the reflective scales. Lastly, the structural mediation modeling was applied to measure the direct and indirect relationship between the constructs used in the research model.

## 3. Results

### 3.1. Demographic Characteristics

Table 1 outlined the demographic characteristics of the survey sample. Of the 1084 respondents, a little less than two-thirds (*n* = 691, 63.74%) belonged to the age bracket of 31–40 years which was followed by those having age up to 30 years (*n* = 196, 18.08%) and age group of 41–50 years (*n* = 182, 16.78%). Only 15 (1.38%) respondents had an age greater than 50 years. There were 701 (64.66%) males and 383 (35.34%) females. As far as their qualification is concerned, most of these respondents were graduates (*n* = 654, 60.33%), followed by postgraduates (*n* = 317, 29.24%), and undergraduates (*n* = 113, 10.42%). In addition, a large majority these respondents had experience 11–15 years (*n* = 728, 67.15%), followed by those in the experience bracket of 6–10 years (*n* = 184, 16.97%) and up to 5 years (*n* = 98, 9.04%). Only 74 (6.82%) respondents had experience greater than 15+ years. Concerning their regional distribution, about one-third each of the respondents belonged to Lahore (*n* = 351, 32.38%), Karachi (*n* = 348, 32.01%), and Islamabad (*n* = 340, 31.36%) Only 29 (2.67%) and 17 (1.56%) respondents belonged to Peshawar and Quetta, respectively.

### 3.2. The Measurement Model

The measurement model analysis was applied for measuring the validity and reliability of the instrument through SmartPLS 3.3.9. Moreover, the structural relations among variables were measured through structural equation modeling (SEM). SmartPLS software is more statistically effective and efficient and less sensitive towards sample size than other software used for covariance-based SEM, such as AMOS [70,71]. This study explored the connection between IL, lifelong learning, creativity, and work performance among the journalists. Before the measuring the relationships between variables, the reliability and validity were ensured of all constructs used in research model (Table 2).

**Figure 2:**
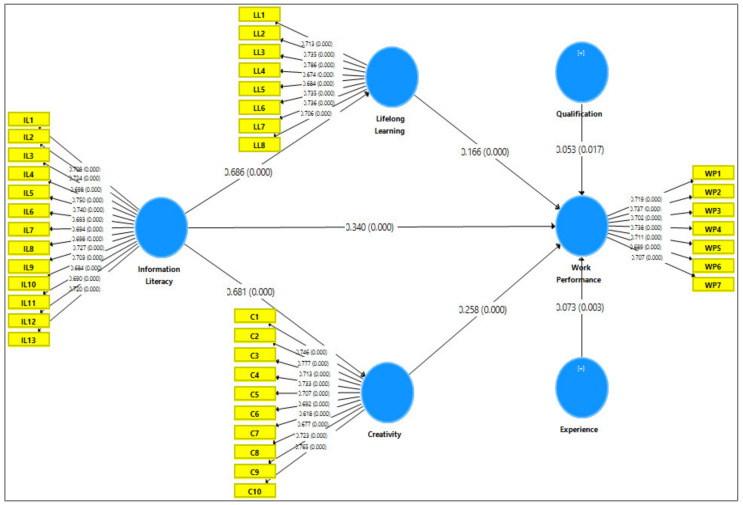
Research Model.

Table 2 indicates the reliability and validity of all measures used in the instrument. The reliability indicators included factor loading, Cronbach’s alpha, and rho_A and composite reliability. The standard value for factor loading is 0.60 [71]. Factor loadings of all items were above 0.60. Therefore, the factor loading of all items were appropriate. Similarly, the Cronbach’s alpha, and rho_A and composite reliability indicators threshold value is 0.70 [70]. Table 2 shows the Cronbach’s alpha, and rho_A and composite reliability indicators having the values above the standard values. It meant that the instrument met the requirement of reliability. The convergent validity was ensured by applying average variance extracted (AVE). The standard value for AVE is 0.50 [72]. Table 2 indicates that AVE of all constructs used in the research model was higher than the threshold value. Therefore, it can be concluded that the instrument used in this study fulfilled the requirements of reliability and validity to collect the data (see Table 2 and Figure 2).

The discriminant validity in partial least squares-structural equation modeling was measured through heterotrait-monotrait (HTMT) approach. This approach was recommended by [73]. They consider that the HTMT approach to measure the discriminant validity in PLS-SEM is more appropriate than [74] approach. They further explained that the [74] approach is an unreliable method to measure the discriminant validity [73]. The HTMT approach is referred as the “item correlation among constructs with the correlations within items of the same construct”. Ref. [73] proposed a less than 0.90 HTMT threshold value which explains that the constructs were proven to be discriminately valid. Table 3 revealed that constructs such as information literacy, lifelong learning, creativity, and work performance met the requirements of discriminant validity.

We ensure that the collinearity issue must be resolved during the structural equation modeling analysis. The Variance Inflation Factor (VIF) technique is applied to resolve this issue. The threshold value for VIF is less than 5 and less than 3 value for VIF is considered ideal [75]. Table 4 indicates that the range of VIF values among dimensions is 1.000 to 2.278, showing no collinearity problems among information literacy, lifelong learning, creativity, and work performance dimensions.

The three main model fit indicators are more usually employed in PLS-SEM analysis to measure the overall fitness of model. These indices are SRMR, NFI, and RMS_ theta. The threshold value for SRMR is less than 0.08. The SRMR value in Table 4 is 0.042 which means that the model was well fit. Similarly, the NFI ideal threshold value is 1. The near to 1 value is considered better model fit. Table 4 indicates that the NFI value is 0.870 which is acceptable and considered a better fit of model. The RMS_theta indices are used for reflective measurement modeling. The threshold value for RMS_theta indices is less than 0.12. Table 4 indicates that the RMS_theta value is 0.077, which means that the model is appropriate. As a result, the model used in this study was shown to be pretty well suited in general. Table 4 displays the collinearity analysis and model fit.

The explanatory power of the model is measured through the R2 value. The range of R2 value is between 0 and 1. The greater the R2 value, the greater the explanatory power. The threshold value of the model up to 0.25, 0.50, and 0.75 are considered weak, moderate, and strong for explanatory power. Table 5 shows that latent variables such as lifelong learning (R2 value, 0.471), creativity (R2 value, 0.463), and work performance (R2 value, 0.482) have moderate level explanatory power.

The explanatory impact of exogenous variables on endogenous variables can be detected through f2 approach. The threshold value range for f2 is between 0.02 < f2 _ 0.15, 0.15 < f2 _ 0.35, and f2 > 0.35 which means that the effect can be categorized as small, medium, and large effect. Table 6 explains that the explanatory effect f2 value of information literacy to creativity is 0.863. This indicates a larger effect. Similarly, the explanatory effect f2 value of information literacy to lifelong learning is 0.889. It also indicates a larger effect. Likewise, the explanatory effect f2 value of information literacy to work performance is 0.098. This indicates a smaller effect. Additionally, the explanatory effect f2 value of lifelong learning to work performance is 0.024. This indicates a smaller effect. Finally, the explanatory effect f2 value of lifelong learning to work performance is 0.057. This also indicates a smaller effect.

### 3.3. Descriptive Analysis

The descriptive statistical analysis was used to measure the levels of IL, lifelong learning, creativity, and work performance of the participants. All scales were divided into five levels (1–5) from lowest to highest. Participants had a high level of information literacy, lifelong learning, creativity, and work performance with a range of mean scores from 4.117 to 4.397. The mean of scores indicates that participants had a high level of perception about their information literacy, lifelong learning, creativity, and work performance.

### 3.4. Structural Model

In this study, the researchers used SmartPLS statistical software to measure the connection between variables used in this study. PLS is a variance-based SEM technique that facilitates the concurrent assessment of the measurement model. This approach analyzes the reliability, validity, and structural and hypothesized relationships among the constructs used in research model. Table 7 indicates the direct and indirect influence of information literacy on work performance.

Furthermore, IL has a positive and significant connection with work performance (β = 0.340, *p* < 0.05), which approved hypothesis H1. Similarly, IL has a positive and significant association with lifelong learning (β = 0.686, *p* < 0.05), which accepted hypothesis H2. Likewise, IL has a positive and significant association with creativity (β = 0.681, *p* < 0.05), which accepted hypothesis H3. Furthermore, lifelong learning has a positive and significant connection with work performance (β = 0.166, *p* < 0.05), which approved hypothesis H4. Additionally, creativity has a positive and significant connection with work performance (β = 0.258, *p* < 0.05), which supported hypothesis H5 (See Table 8 and Figure 2).

### 3.5. Mediating Effect

For the indirect relationship, we measured the relationship between IL and work performance through lifelong learning and creativity. First, we explored the mediating role of lifelong learning in the relationship between IL and work performance (β = 0.144, *p* < 0.05), which approved hypothesis H6. Second, we explored the mediating role of creativity in the relationship between IL and work performance (β = 0.176, *p* < 0.05), which supported hypothesis H7. Moreover, the direct effect of two control variables, namely experience and qualification, on work performance were also measured (β = 0.073, *p* < 0.05, β = 0.053, *p* < 0.05). Both control variables of experience and qualification had a direct and positive connection with work performance (See Table 8 and Figure 2).

## 4. Discussion

This research examined the effects of information literacy on work performance through lifelong learning and creativity among journalists in Pakistan. The research model was proposed based on the existing literature and tested empirically based on data analysis. The descriptive analysis showed that these journalists perceived themselves as information literate, lifelong learners, creative and work performers (Table 6). These findings appeared quite logical as the journalists could not perform well with insufficient capabilities for IL, lifelong learning, and creativity and they continuously engage in unlearning, learning, and relearning at workplace [76,77].

A closer look at the analysis indicated that IL had a direct and positive effect on work performance. These results were generalizable among other developing countries such as China, India, Bangladesh, etc. as they shared similar characteristics with Pakistan. However, more empirical evidence was needed to not only support but also generalize these research outcomes. The results were consistent with the findings of the previous studies showing that IL has a positive connection with work performance [12,45,78]. Similarly, Ref. [2] explored the IL effect on innovation ambidexterity at workplace. The results extracted through applying SEM modeling indicated that the IL of company’s CEOs in Finland enhances the innovation performance. Furthermore, Ref. [79] conducted a study on library personal for measuring the connection between IL and task performance and concluded that IL is a predictor of task performance among library staff. However, this finding contradicted with that of Wu (2018) who reported that IL did not affect directly on work performance. It is plausibly suggested that the employers needed to make arrangements for IL education in the workplace to enhance work performance.

The results also indicated that IL has positive and significant influence on lifelong learning (H2). These results were similar to those of the previous studies that IL has a positive relationship with lifelong learning [49,80]. Likewise, Feng and [22] conducted a study on universities teachers in Fujian Province, China, and the results indicated that IL has a positive and significant relationship with lifelong learning among teachers. Similarly, Ref. [34] conducted a study on 127 physical education and sports teacher candidates; the results of this study found that IL has a positive and significant relationship with lifelong learning skills. Therefore, it may be concluded that the higher IL capabilities enhance lifelong learning in the workplace.

The effect of IL on creativity were also investigated. The results indicated that the IL had a positive and significant effect on creativity (H3). The findings of the current study are consistent with the outcomes of the previous studies that IL on creativity among [12,81]. Similarly, Ref. [23] conducted a study on employees to measure the correlation between IL and creativity. The outcomes of that study indicated that IL has positive and significant relationship with employee creativity. Moreover, other studies from the workplace context also explained the positive influence of IL on creativity [23,51,52,82].Thus, the higher IL competence increases creativity skills in the workplace context.

The direct influence of lifelong learning on work performance was also examined. The results indicated that lifelong learning has a positive and significant connection with work performance (H4). The results of the present study are not differentiated from the previous studies that lifelong learning has a positive effect on work performance [60,83]. Similarly, Ref. [84] conducted study on undergraduate students to measure the students’ lifelong learning characteristics role in their [34] organizational socialization and subsequent extra-role performance. The results indicated that lifelong learning characteristics among students has a positive and strong role to enhance their organizational socialization and subsequent extra-role performance. Likewise, Ref. [53] explained that the role lifelong learning mindset is positive with supervisor-rated performance and job satisfaction, work engagement, and job-related self-efficacy. Hence, the lifelong learning skills enhance work performance.

The direct and positive effect of creativity with work performance were also found in this study (H5). The results of previous studies also affirmed creativity as a predictor of work performance [12,85]. Similarly, Ref. [86] used employees’ creativity to create values for their multiple stakeholders to adapt to the ever-changing and competitive environments. Additionally, a few other studies also explained the positive relationship between creativity and work performance [58,59]. Thus, it may be concluded that the creativity skills also improve work performance.

The mediating role of lifelong learning and creativity in the relationship between IL and work performance were also examined. The results indicated that lifelong learning and creativity played a very significant mediating role in relationship between IL and work performance respectively (H6 and H7). The mediating role of lifelong learning in the relationship between IL and work performance had been investigated for the first time which needed to be supported through future investigations. However, the finding with regard to the mediating role of creativity in the relationship between IL and work performance corroborated the results of [12] who reported similar results in the workplace context. A few other studies also explained that creativity played a mediating role in the relationship between the relationships of employee engagement and intrinsic motivation with job performance [63,64]. Hence, it is concluded that the IL lays the foundations for lifelong learning and creativity which further enhance work performance.

### 4.1. Conclusions and Implications

The proposed research model was conceptualized based on insights drawn from an extensive review of existing literature in the areas of IL, lifelong learning, creativity, and work performance. The results affirm the connection between IL, lifelong learning, creativity, and work performance in the journalists’ workplace. The following conclusions can be drawn. Firstly, IL can confidently be associated with and predicts positively lifelong learning, creativity and work performance. Secondly, lifelong learning and creativity also predict positively work performance. Lastly, IL also has an indirect but positive effect on work performance through the mediating role of lifelong learning and creativity. These results generate pragmatic insights for employers about the effectiveness of IL in the workplace and the way it can influence organizational performance to achieve a sustainable competitive advantage.

The results have implications for educationists, informationists, practitioners, and researchers. First, the enterprises must identify employees with low IL levels and make arrangement for information literacy education. Second, the existing curriculum for trainings in organization needed to incorporate contents for IL, lifelong learning, and creativity skills so that the capacity of workforce might be developed in this regard. Third, the credit-bearing mandatory course for IL education might be incorporated in the existing curriculum at universities so that the capacity building of the prospective workforce might be initiated. Last, special librarians need to collaborate with organizational training teams to plan an integrated IL instruction program in the workplace.

### 4.2. Limitation and Future Research Directions

This research used a self-assessment method for the measurement of IL, lifelong learning, creativity, and work performance. In the self-assessment method, the survey participants overestimate their skills relative to their actual ones which may be the primary limitation. Furthermore, the recruitment of survey participants was made using non-probability convenient sampling method. Therefore, the sample may not be representative of actual population despite the large sample size. In addition, the study was conducted in the context of journalists’ workplace in Pakistan which may limit the generalizability of the conclusions: empirical evidence from other workplace contexts in varied geographical and cultural locales is needed to strengthen the study outcomes. In future research directions, this research may be replicated in other workplace contexts. Future investigations may examine the relationship between information anxiety (IL absence) and work performance, considering lifelong learning and creativity as mediating variables.

## Figures and Tables

**Table 1:** Demographic profile of the participants (N = 1084).

Characteristics	Categories	Frequency (*n*)	Percentage (%)
Age (Years)	Up to 30	196	18.08
	31–40	691	63.74
	41–50	182	16.78
	50+	15	1.38
Gender	Male	701	64.66
	Female	383	35.34
Qualification	Undergraduate	113	10.42
	Graduate	654	60.33
	Postgraduate	317	29.24
Experience (Years)	Up to 5	98	9.04
	6–10	184	16.97
	11–15	728	67.15
	15+	74	8.94
Region	Lahore	351	32.38
	Karachi	347	32.01
	Peshawar	29	2.67
	Quetta	17	1.56
	Islamabad	340	31.36

**Table 2:** Reliability and validity analysis.

Scales	Factor Loading	Cronbach’s Alpha	rho_A	Composite Reliability	Average Variance Extracted (AVE)
Information Literacy (IL)		0.918	0.918	0.93	0.504
IL1	0.708				
IL2	0.724				
IL3	0.698				
IL4	0.750				
IL5	0.740				
IL6	0.693				
IL7	0.694				
IL8	0.698				
IL9	0.727				
IL10	0.703				
IL11	0.684				
IL12	0.690				
IL13	0.720				
Lifelong Learning (LL)		0.868	0.87	0.897	0.521
LL1	0.713				
LL2	0.735				
LL3	0.786				
LL4	0.674				
LL5	0.684				
LL6	0.735				
LL7	0.736				
LL8	0.706				
Creativity		0.894	0.898	0.913	0.513
C1	0.746				
C2	0.777				
C3	0.713				
C4	0.733				
C5	0.707				
C6	0.692				
C7	0.618				
C8	0.677				
C9	0.723				
C10	0.765				
Work Performance (WP)		0.841	0.842	0.88	0.511
WP1	0.719				
WP2	0.737				
WP3	0.702				
WP4	0.738				
WP5	0.711				
WP6	0.689				
WP7	0.707				

**Table 3:** Discriminant validity.

Constructs	Creativity	Information Literacy	Lifelong Learning	Work Performance
Creativity	0.716			
Information Literacy	0.681	0.71		
Lifelong Learning	0.679	0.686	0.722	
Work Performance	0.607	0.639	0.578	0.715

**Table 4:** Collinearity and model fit.

Dimensions	C-VIF	LL-VIF	WP-VIF	Model Fit	
Creativity (C)			2.245	SRMR	0.0420
Information Literacy (IL)	1.000	1.000	2.278	NFI	0.870
Lifelong Learning (LL)			2.249	RMS_Theta	0.077

**Table 5:** R Square.

Variables	R Square	R Square Adjusted
Lifelong Learning	0.471	0.47
Creativity	0.463	0.463
Work Performance	0.482	0.480

**Table 6:** F Square.

Variables	Creativity	Lifelong Learning	Work Performance
Information Literacy	0.863	0.889	0.098
Lifelong Learning			0.024
Creativity			0.057

**Table 7:** Descriptive statistics.

Variables	N	Minimum	Maximum	Mean	SD
Information Literacy	1084	1	5	4.117	0.69315
Lifelong Learning	1084	1	5	4.393	0.71862
Creativity	1084	1	5	4.278	0.71199
Work Performance	1084	1	5	4.120	0.60987

**Table 8:** Direct and indirect relations.

Hypotheses	Direct Relations	Coefficients	Mean	SD	t	*p* Values	Decision
H1	Information Literacy → Work Performance	0.340	0.338	0.049	6.967	0.000	Supported
H2	Information Literacy → Lifelong Learning	0.686	0.687	0.026	26.426	0.000	Supported
H3	Information Literacy → Creativity	0.681	0.683	0.021	32.912	0.000	Supported
H4	Lifelong Learning → Work Performance	0.166	0.166	0.044	3.785	0.000	Supported
H5	Creativity → Work Performance	0.258	0.262	0.041	6.282	0.000	Supported
**Hypotheses**	**Indirect Relations**	**Coefficients**	**Mean**	**SD**	**t**	***p* Values**	**Decision**
H6	Information Literacy → Lifelong Learning → Work Performance	0.114	0.115	0.032	3.612	0.000	Supported
H7	Information Literacy → Creativity → Work Performance	0.176	0.179	0.028	6.286	0.000	Supported
**Control Variables**	Experience → Work Performance	0.073	0.072	0.024	3.024	0.003	Supported
	Qualification → Work Performance	0.053	0.054	0.022	2.383	0.017	Supported

## Data Availability

The data can be provided at request.
